# Adapting and testing an eLearning resource for professionals to support families when a significant caregiver for children is dying with cancer

**DOI:** 10.1186/s12904-024-01601-5

**Published:** 2024-11-21

**Authors:** Carla O’Neill, Jeffrey R. Hanna, Sarah Sheehan, Tanya McCance, Amanda Drury, Cherith J. Semple

**Affiliations:** 1 School of Nursing, Midwifery and Health Systems, University College Dublin, Dublin, Ireland; 2 Institute of Nursing and Health Research, Ulster University, Belfast, UK; 3 South Eastern Health and Social Care Trust, Ulster Hospital, Dundonald, UK; 4 School of Nursing, Psychotherapy and Community Health, Dublin City University, Dublin, Ireland

**Keywords:** Supportive care, Health care professionals, Social care professionals, Children, Adults, Cancer, Digital health, Educational intervention, End of life, Person-based approach

## Abstract

**Purpose:**

Health and social care professionals (professionals) often lack knowledge, skills and confidence to support adults at end of life with significant caregiving responsibilities for children, < 18. A recent systematic review highlighted a dearth of educational interventions (*n* = 2) to equip professionals to provide supportive care to families when a parent has cancer. Addressing an evident gap in education, this paper details the adaption and optimisation of a face-to-face educational intervention to an accessible eLearning resource.

**Methods:**

Guided by the ‘Person-based Approach’, a theory-driven and evidence-based face-to-face educational intervention was adapted and optimised as an eLearning resource. This incorporated current evidence, alongside insights from an expert group, learning technologists and research team, leading to the design of an eLearning prototype. This was optimised for acceptability and usability using think-aloud interviews with end-users (*n =* 13) and patient and public involvement (*n =* 4).

**Results:**

An iterative adaption and optimisation process enabled implementation of navigational improvements, changes to enhance clarity on language and appropriateness of images and interactive components. During optimisation, positive feedback was reported; especially regarding the ‘look and feel’ and on the educational videos and reflective exercises embedded throughout the eLearning resource.

**Conclusion:**

The systematic adaption and optimisation of this novel eLearning resource has aimed to promote relevance, appropriateness, and applicability of an accessible evidence-based and theory-driven training resource for professionals. It has the potential to promote family-centred supportive end of life cancer care, which ultimately can promote better bereavement outcomes. An evaluation of the intervention is required to explore impact on practice.

**Supplementary Information:**

The online version contains supplementary material available at 10.1186/s12904-024-01601-5.

## Introduction

Families are often uncertain about how best to prepare children (< 18) for the end of life experience of a significant adult caregiver, such as a parent [1]. Parents want and need support and guidance to navigate these conversations with their children [1]. Alongside this, children desire to be informed and involved in the end of life experience of a significant adult caregiver [2]. Sadly, many children (< 18) are not prepared for this experience, predisposing them to increased risk of adverse outcomes in bereavement and later life [3]. This includes issues in maintaining and sustaining trusting relationships, a decline in education, as well as increased criminality and involvement with psychiatry [3–5]. Open and honest adult-child communication at end of life has the potential to mediate for these adverse outcomes and promote psychosocial adjustment for the whole family [2].

Health and social care professionals (professionals) are ideally placed to support adults at end of life with cancer to prepare and support their children for this experience [2, 3]. Professionals often feel ill-prepared about providing this important aspect of supportive care, thus requiring training and educational support [3, 6]. A recent systematic review highlighted there are only two educational interventions available to equip professionals in the provision of this important aspect of family-centred cancer care [7–9]. While both educational interventions showed promise in increasing professionals’ confidence to provide family-centred cancer care in practice, there is an inherent need for more accessible educational interventions in this area of supportive care [7].

Cognisant of this gap, a recent face-to-face, evidence-based, and theory-driven educational intervention has been developed for professionals on how best to support parents at end of life with cancer regarding their children [10]. An evaluation of this intervention highlighted an increase in professionals’ perceived self-efficacy toward supporting parents at end of life, with clinicians reporting new approaches and skills on how to have emotive conversations with parents about their children [10]. A more sustainable approach to the provision of this education is necessary to promote global accessibility.

There is a growing recognition internationally of the important role that digital technologies play in health and social care [11, 12]. The All-Ireland Digital Capability Framework for Health and Social Care highlights that digital interventions can improve health and social care services through innovative means to ensure professionals advance person-centred care [13]. Evidence suggests that online learning is just as or more effective for learning than offline methods for professionals and students in health and social care [13, 14]. eLearning offers opportunities to individuals who may face barriers to attending face-to-face training, such as associated costs and logistical difficulties [14]. In addition, eLearning offers a cost-effective means of delivering education [15].

The aim of this paper is to detail the adaptation and report findings from the optimisation of a face-to-face educational intervention to an eLearning resource using the ‘Person-based Approach’ [16].

## Methods

### Adaptation of a face-to-face educational intervention to an eLearning resource prototype

The face-to-face educational intervention comprised of a two-hour session, informed by empirical research [1, 17, 18] and included a communication framework [19], co-produced educational videos roleplaying good practice, and the lived experience of a bereaved parent navigating the end of life experience. Further details on the content and the evaluation of the face-to-face educational intervention have been published elsewhere [10].

The process of adapting and optimising the face-to-face educational intervention to an eLearning resource was guided by the ‘Person-based Approach’ [16]. The ‘Person-based Approach’ is a widely used digital healthcare intervention framework and considers the perspectives of end-users and public and patient involvement (PPI) as integral in the planning, designing and user-testing of the intervention [16].

Guided by the ‘Person-based Approach’ [16] a logic model was developed outlining mechanisms of how the eLearning resource would influence and meet the intended outcomes [16]. Social Cognitive Theory underpinned the logic model, with self-efficacy identified as the primary outcome [20] (See Table 1). Alongside this, ‘Guiding Principles’ were developed which involved: (1) outlining the main design objectives required for the eLearning resource in terms of behaviour change, and (2) the key features of the eLearning resource that would be required to achieve each objective [16, 19].

**Table 1:** Logic model

Situation	Resources	Activities	Outputs	Short- and long-term outcomes/impact
1-in-18 children experience the death of a significant caregiver before adulthood. Children less prepared for the death of a significant caregiver are at greater risk of adverse outcomes. Families want and need guidance from health and social care professionals on how best to support children for the death of an adult with cancer who has significant caregiving responsibilities for children. There is a dearth of available evidence-based educational interventions to equip health and social professionals in the provision of this aspect of care. An evaluation of a recently developed evidence-based and theory-driven face-to-face educational intervention increased professionals’ perceived self-efficacy toward supporting this population at end of life. Sustainable delivery of this education is necessary.	Funding. Time. PPI representation. Expert group. Research team with relevant expertise. Technology *- IT* *- Host platform of eLearning resource* Professional networks and cancer charity organisations. Health and social care professionals for think-aloud interviews (*n* = 10–15) Learning educationalists. Facilities to record video resource(s)	The activities to the project are aligned to the ‘Person-based Approach’ to intervention adaptation and optimisation. Establishment of expert group and PPI representatives, with ongoing consultation and involvement in all activities. Systematic review of existing educational interventions. Development of guiding principles to inform the eLearning resource. Informed by the expert group, PPI representatives, systematic review, the face-to-face educational intervention, and underpinned by Family Resilience Theory and Social Cognitive Theory. Instructional design day with learning educationalists and research team. Development of the eLearning resource prototype with learning educationalists. Optimising the eLearning resource using ‘think-aloud’ interviews with health and social care professionals. Promotion of eLearning resource.	A sustainable, evidence-based, and theory-driven eLearning resource for health and social care professionals, equipping them with the knowledge and skills on how best to support families at end of life who have significant caregiving responsibilities for children, < 18. Peer-reviewed publications in academic journals. Established networks and collaborations with members of the expert group and PPI representatives.	Increased knowledge and greater awareness for health and social care professionals on the experiences, challenges, and support needs of families when an adult is at end of life with cancer who has significant caregiving responsibilities for children. Improve health and social care professionals’ skills, knowledge, and self-efficacy, promoting how best clinicians can support adults at end of life with cancer regarding the children, reducing professional burnout. Better family-cohesion, mental health (adult and child) and educational outcomes for children in bereavement with less input with psychiatry. Subsequently, reducing the burden faced by over-stretched family support services. Contribution to advanced communication skills training on how best to support families at end of life regarding the children, < 18. Policy guidelines on the importance of providing psychosocial supportive care to families when an adult is at end of life cancer, regarding significant caregiving responsibilities for children. Inform educational curricula for pre- and post-registration students from a range of health and social care disciplines on best practice surrounding end of life family-centred communication. Transferability of learning to other life-limiting conditions, and providers of educational and pastoral care.

The content of the face-to-face intervention was then mapped on to Microsoft PowerPoint slides, using a storyboard approach, to include the co-produced video resources and the ‘Talking, Telling and Sharing Framework: End of Life’ [21]. The content was reviewed and refined by the research team, who are nurses and researchers working in clinical and/or academic roles related to cancer care. The project team then collaborated with a team of learning technologists to develop the prototype of the resource. This included careful consideration regarding the right authoring tool for accessibility, being cognisant of universal design learning (UDL) principles [22]. Through discussions with the learning technologists, interactive elements were included to the resource prototype, such as flip-card activities. The decision was made not to include validation items within the resource to reduce user-burden. The prototype underwent iterations between the project team and the learning technologists over a five-month period. Changes made during these iterations predominantly related to the ‘look and feel’ of the resource, due to significant challenges in identifying suitable and appropriate visuals and colours for the resource given the sensitive nature of the topic.

At key points during the adaptation and design phase the research team collaborated with an expert steering group, to gain wider consensus and ensure the resource was relevant, appropriate, feasible and applicable. The expert group comprised of a bereaved parent and teenager as patient and public involvement (PPI) representatives, as well as a senior clinical psychologist, family support worker, computer scientist and a senior cancer nursing academic. A key modification to the prototype following review by the expert group included a shift in terminology from ‘parent’ to ‘adult’ to reflect diversity in significant caregiving responsibilities for children. Reflective activities were also added without forced responses to encourage reflection but not to add additional user-burden.

Following a five-month period, adapting and designing the eLearning resource the final prototype consisted of four sections, with three of the sections reflective of key landmarks during the end of life trajectory for many families: (1) introduction, (2) supporting adults at the time of receiving the poor prognosis, (3) preparing for the future, and (4) navigating the final weeks and days of life. A key focus in this design phase was identifying appropriate visual representation that accounted for the sensitive and emotive topic and diversity of family units [23]. See Table 2 for the learning outcomes of the resource and content overview of each section. The eLearning resource can be accessed by registering a free account on fccceoled.com.

**Table 2:** Learning outcomes of each section of the eLearning resource

Section	Learning outcomes
eLearning resource	• Recognise the support needs of families when an adult with significant caregiving responsibilities for children is at end of life. • Understand the importance of telling children a significant adult is at end of life. • Recognise and reflect on the importance of your role as a professional to support families at end of life
Introduction	• Explore the challenges and needs of: ◦ Professionals in supporting adults at end of life regarding their significant caregiving responsibilities for children (< 18), ◦ Adults who are at end of life with and have significant caregiving responsibilities for children (< 18), ◦ Children (< 18) when a significant adult caregiver is at end of life with cancer. • Improve professionals’ awareness of this aspect of supportive cancer care
Supporting adults at the time of receiving the poor prognosis	• Demonstrate good practice surrounding difficult end of life conversations between professionals and adults. • Use the ‘Talking, Telling, Sharing Framework: End of Life’ to equip professionals to empower adults on when and how to tell the children about a poor cancer prognosis.
Preparing for the future	• Highlight key advice and guidance professionals should provide adults with, as they prepare children and the family for end of life, and the subsequent bereavement period.
Navigating the final weeks and days of life	• Provide advice for professionals on how best to support families in the final weeks and days of life. • Educate professionals on children’s developmental understanding and reactions to death.

### Study design for optimisation of eLearning resource prototype

In line with the ‘Person-based Approach’, qualitative data was collected and analysed iteratively to optimise the eLearning resource [16]. One-to-one think-aloud interviews were conducted remotely, where participants worked through the eLearning resource verbalising their thoughts [16]. An iterative approach to data collection and analysis was utilised to enact changes to the resource [16].

### Participants

Individuals were eligible to participate if they were a registered professional involved in the care of families impacted by cancer at end of life. Participants were recruited through a poster advert, which was promoted and disseminated through social media, hospices, and the research team’s professional networks. Also, the email addresses of the professionals who completed the face-to-face educational intervention and provided consent to be contacted again regarding ongoing research studies in the family-centred cancer care programme of work (ulster.ac.uk/fccc).

### Procedure

Interested and willing professionals contacted JRH via email, who provided them with a Qualtrics link to a participant information sheet and consent form. This provided individuals with details about why the study was being conducted, the research team, what participation would entail, risks involved and who to contact regarding questions on the study. JRH then contacted those who completed the online consent form via email to arrange a suitable time for the interview.

Think-aloud interviews were conducted by JRH, who has experience of qualitative interviewing in this subject area. The topic guide for the think aloud interviews was developed for the purpose of this research. The topic guide was developed by JH and peer-reviewed by the other team members (CS, CON, AD, TMcC, SS). The topic guide is available as a supplementary file [Supplementary file 1]. During the interviews, participants were asked to verbalise thoughts as they worked through the eLearning resource. JRH asked probing questions to facilitate participants to express their reflections on the resource, such as ‘tell me what you are thinking about at the moment’. Interviews were conducted on MS Teams between June and September 2023 and lasted between 32 and 85 min (mAvg = 45-minutes). Interviews were video- and audio-recorded with a direct link to the prototype shared with the participants so they that they could navigate through the resource at their own pace. Participants shared their screens as they worked through the resource enabling observations and reactions to be captured.

### Data analysis

Think-aloud interviews were conducted over three iterative cycles, with approximately four interviews per cycle. Data analysis began after the first cycle of think-aloud interviews using an iterative approach [16, 24, 25]. In line with this approach, the process moved between data collection, analysis, making modifications to the resource and then continuing data collection.

Interviews were transcribed using MS Teams live transcription feature and verified by JRH by listening back to the recording. Data was extracted from the interviews using an extraction sheet in Microsoft Word developed by the research team. Using a content analysis approach, data were extracted relating to the positive and negative comments to the four sections of the resource. Where appropriate, quotes were extracted from the data to provide supporting evidence of the comment.

The project team met after each cycle and decided what modifications should or should not be made to overcome the barriers identified through the data analysis process. The decision process was guided by the Must have, Should have, Could have, Would like (MoSCoW) criteria, which is a prioritisation model to enable the modifications to be sorted by priority [25]. The ‘Person-based Approach’ guiding principles and the Guiding Principles for the intervention also guided decisions about modifications of the resource [25].

Where consensus was not achieved on adoption of modifications as a team, changes were not made, but probed in further interviews to identify what changes, if any, were required. Using the MoSCoW criteria as a guide, the team agreed that no further cycles of interviews were required after the third cycle. This decision was made as the third cycle did not identify any changes that were likely to impact behaviour change, with no feedback categorised as ‘must have ‘or ‘should have’ on the MoSCoW prioritisation model [25]. This decision was also informed by the purposeful sampling method used in the recruitment of participants, with the research team agreeing that an appropriately diverse range of professional and personal experiences were captured within the three refinement cycles.

### Patient and public involvement (PPI)

PPI was integrated across the three iterative cycles. Individual feedback was also collected from expert steering group members at each iterative cycle. Using a proforma table, which was developed for the purpose of this research, in Microsoft Word, these individuals could provide positive and negative comments to each section of the eLearning resource. The proforma table is available as a supplementary file [Supplementary file 2]. Identified from a relevant cancer charity, feedback on the resource was also obtained from a bereaved dad who met with JRH online, as well as collective feedback from two bereaved teenagers who met face-to-face with CJS and JRH. PPI feedback was integrated with the data analysis from the think-aloud interviews at each iterative cycle.

### Ethical considerations

Participants were informed that all information shared would be confidential. Data was stored on Ulster University’s OneDrive and password protected accessible only by the research team. Data protection procedures are in place to destroy all data in accordance with GDPR and Data Protection Act 2018. Ethical approval was obtained from Ulster University [Ref: FCNUR-23-002] and University College Dublin [Ref: LS-22–65].

## Results

In total 20 individuals provided feedback to the resource, comprising of 13 professionals who took part in a think-aloud interview, as well as 3 expert group members and 4 PPI representatives who provided individual or collective feedback (see Table 3 for sample characteristics).

**Table 3:** Characteristics of individuals involved in the user-testing phase

**Think-aloud interview participant characteristic**	*n*	**PPI / expert group representative characteristic**	*n*
**Professional role per cycle**		**Role per cycle**	
*Cycle 1*		*Cycle 1*	
Clinical nurse specialist (oncology)	1	Computer scientist (expert group)	1
Bereavement co-ordinator	1	Subject expert in cancer care (expert group)	1
Social worker (palliative care)	1	Bereaved parent (mum) (PPI)	1
Consultant (oncology)	1	*Cycle 2*	
*Cycle 2*		Clinical psychologist (expert group)	1
Healthcare chaplain	2	Bereaved parent (dad) (PPI)	1
Lead nurse (community)	1	*Cycle 3*	
Service improvement facilitator (palliative care)	1	Bereaved teenager (PPI)	2
District nursing manager	1		
*Cycle 3*			
Ward manager	1		
Consultant (palliative care)	1		
Social worker	1		
Clinical nurse specialist (oncology)	1		
**Years’ professional experience**			
0–10 years	0		
11–15 years	5		
16–20 years	2		
21–25 years	2		
26–30 years	0		
31–35 years	3		
36–40 years	1		
**Location**			
UK (England, Northern Ireland)	11		
Republic of Ireland	2		
**Gender**			
Female	10		
Male	3		

Changes to the resource prototype in cycle one mostly related to navigational difficulties experienced by users during this phase. Cycle two changes predominantly related to clarity on language and terminology throughout the resource. Cycle three provided feedback on prior issues raised in previous cycles that were not mutually agreed upon by the research team. Findings are discussed under three themes: (1) user-experience of the resource, (2) appropriateness of language and terminology, and (3) educational content. Table 4 provides a summary of ‘must have’ [24] changes to the resource at each cycle.

**Table 4:** Rationale for changes rated as ‘must have’ at each user-test cycle stage

	Cycle 1	Cycle 2	Cycle 3
**Theme one: User experience of the resource**	-Reduced white space between blocks to minimise scrolling within each section and to improve usability. - The instruction at each block was changed from ‘click on the images to learn more’ to ‘click on the ‘+’ icons below to learn more’ to aid synchronicity between the instruction and the action. - The size of the visuals and pop-up boxes were reduced to ensure all information was visible on screen without having to scroll, and promote accessibility of the content. - Font size of headings and sub-headings were increased to separate learning blocks within each section.	- Transcripts were included for each video to aid accessibility for users. Also, for users who may be able to play the video due to factors such as no available audio or undertaking the training in a ‘busy’ environment.	- A clearer instruction was added before each video to read ‘select on the icon to play the video’ to aid synchronicity between the instruction and the action. - A static image was included as the backdrop of each video to enhance the look and feel of the video, rather than a black background with just the icon.
**Theme two: Appropriateness of language and terminology**	- Expansion of the statement ‘avoid euphemisms and confusing terminology’ to ‘avoid euphemisms and confusing terminology such as ‘passed away’, ‘lost’, or ‘gone to the stars’, to provide context and aid clarity for users. - Typos were identified and changed.	- The statement ‘I don’t understand the prognosis myself, so how do I begin to tell my children?’ was changed to ‘I really don’t know how long I am going to live, so how am I supposed to tell the children?’ to reflect the sensitive nature of the topic. - The statement ‘younger children may blame themselves if they do not know the truth’ was changed to ‘younger children may blame themselves if they do not know the truth, such as thinking that they caused the cancer by being naughty’ to aid clarity and understanding. - The statement ‘how or when are you going to die?” was changed to “what if they ask how or when are you going to die?’ to reflect the appropriate question children may ask.	- Initially highlighted in participant feedback in cycle two, clarity and context was added to the following statements: 1) ‘what if I say the wrong thing’ to ‘what if I say the wrong thing and cause harm’. 2) ‘as a professional, routinely check-in with the family to see how they are managing the situation’ to ‘some adults may continue to struggle to share the poor prognosis with the children. Routinely check-in with the family to see how they are managing the situation’.
**Theme three: Educational content**	- An infographic ‘roadmap’ depicting the end of life experience for families was removed as it was considered difficult to understand by participants.	- An introductory video was filmed and included at the opening page by replacing the introductory text to promote connectivity to the resource. - A section on children’s development understanding and reactions to death were included to promote learning. - A prompt was included to all videos (except the introductory video) to inform users of their sensitive and emotive nature. This included inserting the following statement above the videos: ‘please note, the content within this video can be upsetting’. - A one-page PDF of the “Talking, Telling, Sharing Framework: End of Life’ framework was included at end of the resource for users’ to print a physical copy.	- Highlighted in cycle two, participants felt there was a need to provide professionals with advice and guidance on how to support the family if someone did not react or respond well to receiving the poor prognosis. As a result, the following statement was included: ‘some adults and children may require additional help and support as they navigate the end of life period. It can be helpful to find out what support services are available for families and offer onward referral’. - As highlighted by the teenagers involved in user-testing the resource, in the section outlining common reactions by teenagers, the following reaction was added: ‘feeling numb and unable to make sense about their emotions.’

### Theme one: user-experience of the resource

In general, participants positively commented on the look and feel of the resource. The design and colours used were considered appropriate by participants, with many feeling the white background with relevant bold coloured blocks was ‘distraction free’. The visuals (icons and vectors) that were integrated throughout the resource were viewed as suitable, relevant, contemporary, non-controversial and appropriate by professionals, often considered as ‘simplistic enough not to distract from the messages being conveyed’.

*“The feel of the resource is calm. It’s an area that is scary as a professional and yet it just feels calm.’ [Palliative care social worker – cycle one]*.

Alongside participants’ reports, it was observed that individuals found the resource as ‘easy to navigate’. In all cycles, participants stated the progress bar was a useful point of reference for understanding how much of the resource they had completed. This navigational tool was also helpful for self-assessing whether they would have enough time left to complete the resource, or return to it at another time. Participants positively commented on how the resource had no mandatory validation items, so potential users could focus on the content most relevant for their practice. Participants felt the resource was ‘appropriately balanced’ with sufficient flip-card activities and clicking on icons to reveal content to aid connectivity and interactivity.

*“Excellent use of interactive clickable components to sustain your attention to the resource*,* but not too many that you become distracted from the learning” [PPI written feedback*,* computer scientist – cycle one]*.

At cycle one, participants felt the eLearning resource could be improved by reducing the blank space between different blocks within each section to minimise scrolling. Changes were also made to the instructions within the resource to aid synchroneity with the actions. To promote resource usability, transcripts of the videos were included after cycle two for users who may be unable to play the videos and to promote inclusive learning.

*“I am a very visual learner*,* but I appreciate that there are some people who learn by audio. I like how you can listen to the introduction video and still obtain the same messages as those who watch the video*,* as well as those who will read the script.” [Social worker – cycle three]*.

### Theme two: appropriateness of language and terminology

Overall, participants felt that the language used throughout the resource was clear, concise, and accessible.

*“I can see there has been a lot of careful thought and consideration in the language. This is such a sensitive and emotive topic and I think you have conveyed your messages gracefully. What I do like is how the text is clear*,* accessible and is short and to the point.” [Healthcare chaplain – cycle two]*.

*“Sometimes healthcare staff can feel guilty about not using that language or knowing if it’s okay to tell the parents to use those terms. This resource provides that important reassurance to professionals.’ [Palliative care consultant – cycle three]*.

Nonetheless, there were some areas within the resource where terminology was modified to enhance clarity and understanding of the messages being conveyed. This included rephrasing of text such as “*I don’t understand the prognosis myself*,* so how do I begin to tell my children?”* to *“I really don’t know how long I am going to live*,* so how am I supposed to tell the children”.* On occasion, some participants felt examples or additional context was required to enhance clarity, such as explaining what euphemisms should be avoided by adults when explaining death to children. As highlighted in bold, a further example where additional text was required was to the statement “younger children may blame themselves if they do not know the truth, such as thinking they caused the cancer by being naughty”.

### Theme three: educational content

The video resources integrated into the resource were highly regarded by participants across the interview cycles, repeatedly described as ‘poignant’ and ‘powerful’. Unanimously, participants considered the videos roleplaying good practice would be useful and helpful for professionals as they consider having difficult conversations with adults in practice. Consistently, participants considered the videos as ‘effective learning tools’ that would aid connectivity between learning and implementation to practice.

*“That video is excellent. This is good learning*,* and instrumental for professionals on their learning and implementation for practice. So many useful tips here that addresses the questions adults have*,* and*,* well*,* us too as professionals. I can see so many professionals lifting this and using it.” [Palliative care service improvement facilitator – cycle two].*

Participants noted the reflective exercises were necessary after ‘hard-hitting content’ to enable users to reflect the learning on their past and future clinical interactions. While some participants felt there was a need for a reflective exercise in each of the sections, others considered ‘less was more’. Alongside this, some participants felt there was a need for an open-text box for users to document thoughts on the reflective activities; however, most considered this unnecessary as it reduced the user-burden.

*“This reflection is so appropriate. You are walking in the parent’s journey and building up the tools you need on how to support at that particular journey. It’s crucial to stop and take stock of what you have learnt and how you are going to take that back to your practice. This is a very reflective learning moment for me too.” [Oncology clinical nurse specialist - cycle one]*.

Participants reflected on personal and professional experiences of how age-appropriate information must be shared with the children regarding the adult’s declining health, and end of life experience. However, many participants felt that professionals who will be using the resource would benefit from a section outlining understanding children’s developmental understanding and reactions to understanding of death, so they (the professional) can share this information with adults. This section was developed and included after cycle one. In cycles two and three, participant’s positively highlighted this information as useful and relevant to clinical practice.

*“There’s a lot of good stuff here that completely makes sense but I wasn’t consciously aware of it. It’s very important to highlight those developmental milestones. It’s helpful to have that level of information so you can tell parents of the same’. [District nurse manager*,* cycle two]*

A ‘roadmap’ infographic depicting an overview of the end of life experience for families, used in the face-to-face educational intervention, had been included at the end of each section of the eLearning resource. However, in cycle one it was apparent from participants’ reactions that the ‘roadmap’ was difficult to understand, often reported as ‘confusing’. Participants in cycle one highlighted how the ‘remember’ block summarising key information in bullet points was a better presentation of the salient take-home messages, rather than the ‘roadmap’ infographic. In subsequent cycles where the infographic had been removed, participants repeatedly commented on the usefulness and helpfulness of the ‘remember’ section, especially for learners who may not have processed all of the information within the section.

*‘There is a lot of really valuable information in this section and it is so important to encapsulate the key take home messages. Absolutely*,* what you have listed here is exactly what you want people to take away and do in practice. I find this much easier to understand than the picture above.’ [Bereavement coordinator – cycle one]*.

## Discussion

Using a systematic and iterative process, as well as significant input from an expert group and PPI representatives, this project adapted an evidence-based and theory-driven face-to-face educational intervention to a novel, self-directed eLearning resource. Optimised across three user-test cycles with end-users, an expert group and PPI individuals, there were key navigational difficulties resolved and minor changes to language and content to maximise the relevance, usability, appropriateness, and applicability of the eLearning resource.

Where an evidence-based intervention has been positively evaluated, there is greater expectation of effectiveness in adapting the intervention for relevant contexts rather than developing a new intervention [26]. Adaptation and implementation of existing interventions are more likely to save resources, costs and time compared to the development of a new intervention [27]. While intervention adaptation focuses on adapting an intervention for a new context, intervention development involves developing a new intervention [27]. There are situations where a hybrid approach of intervention development and adaptation may be taken and is necessary for researchers to distinguish between both [28]. This study adapted a face-to-face educational intervention, to an online format. The research team were cognisant of the pedagogical literature, in that online learning is more nuanced and complex [29] consequently, the face-to-face content could not be merely replicated into an online format [29] with expertise of educational learning technologists sought at the storyboard development phase. Collectively, clear learning goals and outcomes were established and matched [30] (Table 2); with videos, interactive exercises and reflective activities purposefully developed across four small sections to achieve learning outcomes [31] and retain the attention of the learner [23].

For example, in this study, it was identified that an adapted version of the ‘roadmap’ included in the face-to-face educational intervention was not appropriate for the eLearning resource. Similarly, elements were included to the eLearning resource that were not part of the face-to-face educational intervention, such as reflective exercises. This emphasises the importance of interventions to be adopted with rigorous optimisation with end-users to ensure effectiveness, usability and relevancy [16, 32].

It was identified that a planned and targeted approach to PPI and expert group engagement throughout this project enabled greater flexibility for PPI representatives and expert group members to be involved. This allowed important insights, reflections and perspectives to be captured which complemented the adaptation and optimisation process. In this project, it was important to extend involvement beyond end-users (professionals) to those who would be impacted by the resource in practice (adults and children, pre-and-post bereavement) [33]. Individual and collective feedback from PPI individuals was beneficial in promoting the rigour, relevance and quality of the eLearning resource [33]. Aligned to the ‘Person-based Approach’ [16], PPI input is crucial from inception through to completion of research, emphasising the importance of conducting research ‘with’ rather than ‘for’ people [34]. It is important for research to carefully identify and report on the level of involvement [co-researcher, advisor, personal engagement] and role type of involvement [partnership, collaboration, consultation, participation] at the different stages of the research process [35, 36]. Clear and transparent understanding of the role and involvement of PPI representatives could help toward the inclusion of PPI input to clinical research [37]. Ultimately, this has the potential to promote the effectiveness of research being implemented to practice [16, 33].

The ‘Person-based Approach’ is an internationally recognised model for planning, developing, adapting, optimising, evaluating, and implementing behavioural health interventions for supporting better health-related outcomes [16, 25]. There are many activities within the ‘Person-based Approach’ for developing digital health interventions that are recommended by the recently updated Medical Research Council and INDEX guidance [38]. This includes the importance of stakeholder and PPI input, and optimisation of the intervention [38]. Guided by the ‘Person-based Approach’, this project identified the significance of spending time in the planning and adaptation phases to understand the needs and views of users, leading to fewer problems identified, or the requirement for significant changes in the optimisation phase [16]. It was identified in cycle one that changes to the resource were predominantly related to navigational issues and look and feel of the resource, with more content specific issues identified in cycle two. It can be argued that by the third cycle, the eLearning resource was relevant, applicable, engaging, and likely to be effective in practice.

While the decision was made with PPI representatives to include vectors as visual representation in the eLearning resource, there were significant challenges in identifying visuals that are reflective of cultural diversity, familial set-ups and are sensitive and relevant to the content. This poses the question as to what is appropriate visual representation for use in resources within cancer and palliative care? Although this period created tensions from the digital technologists who had a keen desire to progress to the user-testing phase sooner, in line with the ‘Person-based Approach’ the research team and expert group were cognisant of the need to ensure the eLearning resource prototype was considered relevant and appropriate before progressing to the optimisation stage [16].

This eLearning resource has the potential to promote professionals’ provision of family-centred care to adults at end of life with cancer who have significant caregiving responsibilities for children. This can lead to better mental and physical health outcomes at end of life and in bereavement for adults and children, promoting psychological growth [2–5]. There is a need to evaluate the effectiveness of the eLearning resource to explore its acceptability and useability in clinical practice through the lens of professionals and families, which is the next step of this research project.

### Limitations of the study

There was a lack of cultural and ethnic diversity in the optimisation phase of this eLearning resource. The sample comprised of white participants living and working in the UK and Ireland. This limits the ability to conclude whether the eLearning resource is feasible and useable to non-Western populations and ethnic and cultural minority groups. Several participants of the think-aloud interviews had completed and were recruited through the face-to-face intervention. This may lead to some bias as those participants had previous training in this area and also showed professional and personal interest in this area.

## Conclusion

The systematic and iterative ‘Person-based Approach’ enabled important insights to enhance the content and usability of the eLearning resource. When adapting a face-to-face educational intervention to an eLearning resource, effective instructional design strategies such as clear, small, sub-sections of multiple components using video, graphics, interactive activities and real-life scenarios should be adopted to promote the effectiveness of online learning and satisfaction for health and social care professionals. This novel eLearning resource meets an important identified gap in the provision of evidence-based and theory-driven training which is accessible for professionals; promoting convenience of learners, cost-effectiveness and reach. This eLearning resource has the potential to enhance professional-led support to adults at end of life with cancer who have significant caregiving responsibilities for dependent children. Ultimately, this can promote better outcomes for families pre and post bereavement.

## Supplementary Information

Supplementary Material 1.

Supplementary Material 2.

## Data Availability

The datasets used and/or analysed during the current study are available from the corresponding author on reasonable request.

## References

[1] Hanna JR, McCaughan E, Semple CJ. Challenges and support needs of parents and children when a parent is at end of life: a systematic review. Palliat Med. 2019;33(8):1017–44. 31244381 10.1177/0269216319857622

[2] Wray A, Seymour J, Greenley S, Boland JW. Parental terminal cancer and dependent children: a systematic review. BMJ Support Palliat Care. 2022. 10.1136/bmjspcare-2021-003094. 35091436 10.1136/bmjspcare-2021-003094

[3] Dalton L, et al. Communication with children and adolescents about the diagnosis of a life-threatening condition in their parent. Lancet. 2019;393(10176):1164–76. 30894272 10.1016/S0140-6736(18)33202-1

[4] Ellis J, Dowrick C, Lloyd-Williams M. The long-term impact of early parental death: lessons from a narrative study. J R Soc Med. 2013;106(2):57–67. 23392851 10.1177/0141076812472623 PMC3569022

[5] Niemelä M, Paananen R, Hakko H, Merikukka M, Gissler M, Räsänen S. The prevalence of children affected by parental cancer and their use of specialized psychiatric services: the 1987 Finnish birth cohort study. Int J Cancer. 2012;131(9):2117–25. 22307957 10.1002/ijc.27466

[6] Frerichs W, Geertz W, Johannsen LM, Inhestern L, Bergelt C. Child-and family-specific communication skills trainings for healthcare professionals caring for families with parental cancer: a systematic review. PLoS One. 2022;17(11):e0277225. 36350839 10.1371/journal.pone.0277225 PMC9645618

[7] Sheehan S, Hanna JR, Drury A, McCance T, Semple CJ, O’Neill C. Review of Educational Interventions to Equip Health and Social Care Professionals to Promote End-of-Life Supportive Care when a Parent with Dependent Children is Dying with Cancer. In: Seminars in oncology nursing. Elsevier; 2023. p. 151474. 10.1016/j.soncn.2023.151474. 10.1016/j.soncn.2023.151474 37481410

[8] Dowling M, Shewbridge A, Ryan C, et al. 'Development and Implementation of an Online Education Program on Advanced Breast Cancer for European Cancer Nurses: ABC4Nurses Project: a Brief Report'. J Cancer Educ. 2023. p. 1662–6. 10.1007/s13187-023-02319-3. 10.1007/s13187-023-02319-3 PMC10509077 37336799

[9] Turner J, Clavarino A, Yates P, Hargraves M, Connors V, Hausmann S. Enhancing the supportive care of parents with advanced cancer: development of a self-directed educational manual. Eur J Cancer. 2008;44(12):1625–31. 18375118 10.1016/j.ejca.2008.02.045

[10] Hanna JR, Semple CJ. Mixed-methods evaluation of a face-to-face educational intervention for health and social care professionals to deliver family-centred cancer supportive care when a parent with dependent children is at end of life. Psycho Oncol. 2024;33(7):1–11. 10.1002/pon.6374 38977423

[11] WHO guideline: recommendations on digital interventions for health system strengthening. Geneva: World Health Organization; 2019. Available from:https://www.who.int/publications/i/item/9789241550505. 31162915

[12] Williamson L et al. National Nursing and Midwifery Digital Health Capability Framework. 2020.

[13] Pei L, Wu H. Does online learning work better than offline learning in undergraduate medical education? A systematic review and meta-analysis. Med Educ Online. 2019;24(1):1666538. 31526248 10.1080/10872981.2019.1666538 PMC6758693

[14] Souza CL, Mattos LB, Stein AT, Rosário P, Magalhães CR. Face-to-face and distance education modalities in the training of healthcare professionals: a quasi-experimental study. Front Psychol. 2018;9:1557. 30186214 10.3389/fpsyg.2018.01557 PMC6113380

[15] Bandla H, Franco RA, Simpson D, Brennan K, McKanry J, Bragg D. Assessing learning outcomes and cost effectiveness of an online sleep curriculum for medical students. J Clin Sleep Med. 2012;8(4):439–43. 22893775 10.5664/jcsm.2042 PMC3407263

[16] Yardley L, Morrison L, Bradbury K, Muller I. The person-based approach to intervention development: application to digital health-related behavior change interventions. J Med Internet Res. 2015;17(1):e4055. 10.2196/jmir.4055 PMC4327440 25639757

[17] McCaughan E, Semple CJ, Hanna JR. Don’t forget the children’: a qualitative study when a parent is at end of life from cancer. Support Care Cancer. 2021;29(12):7695–702. 34143326 10.1007/s00520-021-06341-3 PMC8550711

[18] Hanna JR, McCaughan E, Beck ER, Semple CJ. Providing care to parents dying from cancer with dependent children: health and social care professionals’ experience. Psycho-Oncology. 2021;30(3):331–9. 33091180 10.1002/pon.5581

[19] Bingham SL, Semple CJ, Flannagan C, Dunwoody L. Adapting and usability testing of an eLearning resource to enhance healthcare professional provision of sexual support across cancer care. Support Care Cancer. 2022;30(4):3541–51. 35020074 10.1007/s00520-022-06798-w PMC8752582

[20] Bandura A. Social foundations of thought and action. Englewood Cliffs NJ. 1986;23–28:1986.

[21] Semple CJ, McCaughan E, Smith R, Hanna JR. Parent’s with incurable cancer:‘Nuts and bolts’ of how professionals can support parents to communicate with their dependent children. Patient Educ Couns. 2022;105(3):775–80. 34294491 10.1016/j.pec.2021.06.032

[22] C. CAST, Universal design for learning guidelines version 2.0. Author Wakef MA. 2011.

[23] McCall M, Spencer E, Owen H, Roberts N, Heneghan C. Characteristics and efficacy of digital health education: an overview of systematic reviews. Health Educ J. 2018;77(5):497–514.

[24] Bradbury K, Watts S, Arden-Close E, Yardley L, Lewith G. Developing digital interventions: a methodological guide. EvidBased Complement Altern Med. 2014;2014:561320. 10.1155/2014/561320. 10.1155/2014/561320 PMC3932254 24648848

[25] Bradbury K, et al. Using the person-based approach to optimise a digital intervention for the management of hypertension. PLoS One. 2018;13(5):e0196868. 29723262 10.1371/journal.pone.0196868 PMC5933761

[26] Moore G et al. Adaptation of interventions for implementation and/or re-evaluation in new contexts: the ADAPTguidance (v1. 0), ed. 2022.

[27] O’Cathain A, et al. Guidance on how to develop complex interventions to improve health and healthcare. BMJ open. 2019;9(8):e029954. 31420394 10.1136/bmjopen-2019-029954 PMC6701588

[28] Escoffery C, et al. A systematic review of adaptations of evidence-based public health interventions globally. Implement Sci. 2018;13(1):1–21. 30257683 10.1186/s13012-018-0815-9 PMC6158804

[29] Conceição SC, Howles L. Designing the online learning experience: evidence-based principles and strategies. Bloomfield: Taylor & Francis; 2023.

[30] Sinclair PM, Kable A, Levett-Jones T, Booth D. The effectiveness of internet-based e-learning on clinician behaviour and patient outcomes: a systematic review. Int J Nurs Stud. 2016;57:70–81. 27045566 10.1016/j.ijnurstu.2016.01.011

[31] Cook DA, Levinson AJ, Garside S. Time and learning efficiency in internet-based learning: a systematic review and meta-analysis. Adv Health Sci Educ. 2010;15:755–70. 10.1007/s10459-010-9231-x 20467807

[32] Sundell K, Beelmann A, Hasson H, von Thiele Schwarz U. Novel programs, international adoptions, or contextual adaptations? Meta-analytical results from German and Swedish intervention research. J Clin Child Adolesc Psychol. 2016;45(6):784–96. 25864716 10.1080/15374416.2015.1020540

[33] Muller I, et al. Combining qualitative research with PPI: reflections on using the person-based approach for developing behavioural interventions. Res Involv Engagem. 2019;5(1):1–8. 31807316 10.1186/s40900-019-0169-8 PMC6857167

[34] Research NIOH. Patient and Public Involvement. Available: http://www.nets.nihr.ac.uk/ppi.

[35] Colomer-Lahiguera S, et al. Patient and public involvement in cancer research: a scoping review. Cancer Med. 2023. 10.1155/2014/561320. 10.1002/cam4.6200 PMC10417078 37329180

[36] Gray-Burrows KA, et al. Role of patient and public involvement in implementation research: a consensus study. BMJ Qual Saf. 2018. 10.1136/bmjqs-2017-006954. 29666310 10.1136/bmjqs-2017-006954 PMC6166593

[37] Røssvoll TB, Rosenvinge JH, Liabo K, Hanssen TA, Pettersen G. Patient and public involvement in health research from researchers’ perspective. Health Expect. 2023;26(6):2525–31. 37602908 10.1111/hex.13853 PMC10632614

[38] Skivington K et al. A new framework for developing and evaluating complex interventions: update of Medical Research Council guidance. BMJ. 2021;374. 10.1136/bmj.n2061. 10.1136/bmj.n2061 PMC8482308 34593508

