# Supplementary material for: Adapting and testing an eLearning resource for professionals to support families when a significant caregiver for children is dying with cancer
Source: BMC Palliat Care. 2024 Nov 21;23:268. doi: 10.1186/s12904-024-01601-5 (PMC11580503; doi:10.1186/s12904-024-01601-5)
Supplement: Supplementary file 2 — Supplementary Material 2. [file 12904_2024_1601_MOESM2_ESM.docx]

|  | What works well…. | Would be better if…… |
| --- | --- | --- |
| **Welcome to this eLearning resource**  **Content page** | . |  |
| **Section 1: Introduction** |  |  |
| **Module 2: Supporting adults at the time of receiving the poor prognosis** |  |  |
| **Module 3: Preparing for the future** |  |  |
| **Module 4: Navigating the final weeks and days of life** |  |  |
| **Summary and further learning** |  |  |

**Some additional feedback questions below:**

| 1. **What is your overall impression of the eLearning resource? – look, feel, navigational features, images** 2. **What do you feel is pertinent to change within the resource?** 3. **Do you feel something is missing?** 4. **What impact do you feel the resource will have in practice?** 5. **Please add any other comments you feel are relevant to enhance the eLearning resource for professionals.** |
| --- |
